# Abused by the Patriarchy: Male Victims, Masculinity, “Honor”-Based Abuse and Forced Marriages

**DOI:** 10.1177/0886260521997928

**Published:** 2021-02-25

**Authors:** Mohammad Mazher Idriss

**Affiliations:** 1 Manchester Metropolitan University, Manchester, UK

**Keywords:** Domestic violence, predicting domestic violence, male victims, predicting ‘honor’-based abuse, forced marriages, patriarchy, masculinity, assessment, cultural contexts

## Abstract

The causes and effects of what is often referred to as “honor”-based violence/abuse (HBV/A) and forced marriages on men and boys is an under-researched field of patriarchal violence. This lack of research has resulted in an imperfect understanding of how and why men become victims of HBV/A and an absence of an effective theoretical framework in which to analyze their experiences. Through an examination of 29 Case Files obtained through a gender-neutral domestic abuse refuge charity in the East Midlands, the United Kingdom, this original research will explore the ways that men, particularly younger males and those who do not conform to cultural norms of masculinity, are harmed by patriarchal structures. In doing so, this article brings to light new data and adds to the patriarchal framework for understanding HBV/A and why it is committed against men. The results of this study reveal that the ways in which HBV/A and forced marriages were presented in the Case Files present both analogies to and distinctions with the infliction of Violence Against Women and Girls (VAWG) in similar circumstances. This discovery is important for several reasons: (a) it demonstrates that men and boys are harmed by patriarchy and that patriarchal theories of violence must therefore evolve to better recognize groups of male victims; (b) it provides a typological framework to identify the different types of male victims, the types of abuse and the perpetrators involved, including the involvement of mothers as primary and secondary perpetrators; and (c) that male victims require appropriate intervention and must be taken seriously by state agencies if men are to come forward and disclose abuse.

## Introduction

Research in the field of honor-based violence/abuse (hereafter “HBV/A”) and forced marriages has significantly increased over the last two decades, part of a worldwide movement researching Violence Against Women and Girls (hereafter “VAWG;” Gill et al., 2014). It reveals the hidden and unreported nature of such harmful practices, as well as the poor responses to victims seeking intervention (Idriss, 2018a; Mulvihill et al., 2019). Research has played a vital role in uncovering male violence against women, including so-called “honor” killings, where women are killed for allegedly violating cultural norms relating to sexuality and who are perceived to have “tarnished” their family’s reputation. Although previous studies have begun to draw attention to the experiences of male victims of domestic violence, current understanding in this area remains limited (Hine et al., 2020). Even less research has been conducted on male victims of HBV/A and forced marriages and how and why they are affected by patriarchal violence. This is an important area of inquiry because the gender-specific experiences and needs of male victims have been neglected within this field. This article seeks to provide more detailed analysis of men’s experiences and the nature, typology of abuse, and perpetrators these cases present.

### Extending the Categories of Victims of HBV/A and Forced Marriages?

HBV/A and forced marriages involve male perpetrators who are schooled in an everyday culture of patriarchy, which inculcates traditional sex roles, discrimination, and the objectification of women. So-called “honor” killings fall within the definition of femicide as the misogynistic killing of women and represents the brutal end of a broad continuum of VAWG (Gryzb, 2016; Sev’er & Yurdakul, 2001). “Honor” is said to reside within the bodies of women as women are the supposed “repositories” of “honor” (Gill et al., 2014). Within a radical feminist framework, women are expected to protect their sexual “honor” for the duration of their lives. Women must also protect the “honor” of other women related to them, including daughters and granddaughters. While “honor” is related to the chastity of women, it is also considered by the patriarchy to be far too important to be entrusted to women alone. This is an overt demonstration of patriarchy since it is centered upon controlling women’s sexual and reproductive powers by claiming the female body as “man’s territory” (Sev’er & Yurdakul, 2001). Like other forms of VAWG, “honor” killings are nothing more than a conscious process of intimidation by which men keep women in a state of fear (Brownmiller, 1975). Sev’er and Yurdakul state that “the patriarchal culture … [is] … frightened by the emerging sexuality of young women and their (potential) challenge to male rules … cutting down a few women in their prime of their youth is expected to deter other young women from expressing themselves in a sensual way” (Sev’er & Yurdakul, 2001, p. 986).

However, that men may also experience HBV/A or forced marriage if they challenge patriarchal ideology, namely, through the discovery of their homosexuality or their perceived wayward or Westernized behavior, has been historically neglected within academic research. Despite the wealth of literature exploring female victimization, men’s victimization in HBV/A and forced marriage studies have been largely omitted from the discourse. This exclusion does not provide the full picture of victimhood and how a significant minority of male victims become the target of abuse. One explanation for this might be that male victimization is at odds with certain understandings of VAWG—historically, HBV/A and forced marriages have been viewed as a form of abuse perpetrated by men against women and so has been framed as a substantive “women’s issue” within the (valuable) body of work. Activists and academics alike have therefore focused their attention almost exclusively on female victims. However, as an accepted field of study, it is essential that all victims are studied so that appropriate measures and services can be put into place. The lack of studies on male victimization limits scientific knowledge, capturing only the paradigmatic examples (and therefore creating stereotypes), while simultaneously limiting scientific knowledge and understanding of other (minority) groups (Samad, 2010). As the area of study matures, the full range of victims must be considered in order to ensure that appropriate approaches are implemented across the board. In particular, how men may dominate other men is under-developed and under-theorized: theories on HBV/A and forced marriages must now begin to seek explanations for the occurrence of male victimization better than the existing literature has so far developed. The way in which HBV/A and forced marriages have been politically and theoretically constructed over the last two decades has contributed to the invisiblization and marginalization of male victim groups. By ascribing men as perpetrators, the theoretical connections between patriarchy, male perpetrators, and masculinity (Connell, 1995) have become so well established that any conceptualization of men as “victims” (or women as perpetrators) requires one to move outside the traditional boundaries of thinking, all within a climate that has been slow to acknowledge other paradigms.

The (UK government’s) Forced Marriage Unit (FMU, part of the Home Office) on June 25, 2020, detailed that of the 1,355 cases that it dealt with in 2019, 80% concerned women and 19% concerned men (the remaining 1% did not declare their gender; Home Office, 2020); similarly, on May 24, 2019, of the 1,322 cases that the FMU dealt with in 2018, 75% concerned women and 17% concerned men (the remaining 8% did not declare their gender; Home Office, 2019); and on March 16, 2018, of the 1,196 cases dealt with in 2017, 77.8% of cases that the FMU supported concerned women and 21.4% male victims (Home Office, 2018). These statistics are not to be taken to be reflective of prevalence, as the figures do not take into account other organizations that support men, nor does it take into account the under-reported and “hidden” nature of such abuse. Actual figures relating to male victims of forced marriages are likely to be heavily under-reported in relation to prevalence (this is also true of women). However, what is interesting is that the FMU data reveal that over the last three years, on average around 20% of its caseload has specifically concerned men. This sizeable minority demonstrates the existence of male victims within government statistics for some years, despite being ignored and overlooked in the wider literature in general.

If men are victims the assumption is that they have “tarnished” the reputation of a woman and so have been killed as an act of revenge for “dishonoring” the girl’s family (refer to R v Chomir Ali, 2011; Dyer, 2015; R v Tabraz (Mohammed), 2020). However, there are many other types of cases where men may be victims in other circumstances. Men may be viewed “dishonorable” if they refuse to take part in an arranged or forced marriage, or forced into marriage because of their disabilities with families believing that that is the best way to support them in the long term (Clawson and Fyson, 2017). Men may also be forced into marriage “as an antidote for their gayness” (Samad, 2010, pp. 199–200). Due to the stigma associated with being gay in South Asian communities, men may be forced to marry women and emotionally blackmailed into silence for the sake of the family’s “honor” (Jaspal & Siraj, 2011; Siraj, 2006). There is a need for male victims to be conceptualized as a subset of the victims of patriarchy, where similar kinds of domination exist that is the cornerstone of hegemonic masculinity and VAWG (Javaid, 2016b, p. 284).

### Masculinity, HBV/A, and Forced Marriages

There is no one single definition of “masculinity” and theorists often talk about “masculinities”—that is that there are a number of “masculinities” but that there is one central concept—hegemonic masculinity—“against which all other masculinities are measured,” and how one group in society dominates and subordinates another group (Migliaccio, 2001, pp. 67–81). This is important because the concept of masculinity for the communities under discussion in this article demonstrates that such communities take masculinity so seriously that they are prepared to resort to violence in order to enforce those norms. “Hegemony” gives emphasis to the dominance of a main masculine identity, particularly portrayed by means of the media, which Connell explains is socially constructed (Connell, 1987, pp. 183–188; Connell, 2000, pp. 76–80). It is constructed through the likes of television, film, advertising, physical sport, acts of violence, heterosexual activity, and religion (Connell, 2000, pp. 76–85; MacKinnon, 2003). Portraying these forms of “machoism” signifies manliness, power, strength, and a strong sex drive, characteristics that every man should possess (Haywood & Mac an Ghaill, 2003, pp. 67–69; Weiss, 2010). Hegemonic masculinity forces men to comply with gendered expectations, but men from lower classes, gay men, and those from minority groups are all measured against it and are thus labeled as “inferior,” placed outside the mainstream order and accorded a lower status in the social hierarchy (Migliaccio, 2001, pp. 206–207).

“Masculinity … is [also] defined less by what it is and more by what is it not”—and exists in contrast to femininity (Bates, 2005, pp. 67–81; Migliaccio, 2001, pp. 205–206). This approach would regard one of the primary roles of a man is not to behave like or resemble a woman and an association with femininity can emasculate men (Migliaccio, 2001). The fear of being classed as “feminine” may force some men to avoid situations of disclosing personal information or instances that could classify them as “feminine,” such as admitting to their sexual orientation or their experiences of abuse, which calls into question their masculine identity and one which reflects upon them personally (Allen-Collinson, 2009; McCarrick et al., 2016). This may explain why some homosexual men may avoid “coming out” about their sexual orientation, or feign heterosexuality, for fear of being labeled with characteristics that are regarded as effeminate, unmanly, or otherwise have their personal identity threatened (Jaspal & Siraj, 2011; Rogers, 2017). Men and boys are socialized about masculine gender roles, which contributes towards being marginalized in a hegemonic society (Hogan et al., 2012; Tsui, 2014). Masculine notions dictate that men should deny pain, refuse assistance and dominate others, but crossing the gender (feminine) boundary can lead to stigmatization (Cook, 2009, pp. 92–93). Men who are contemplating reporting abuse may fear confirming that they are powerless, undermining dominant and social ideals on masculinity—and it is these issues that have been neglected within this area of study.

The disciplines of criminology and victimology have been accused of being “gender-blind” when concerning male victims in other areas too, such as male rape and sexual assault (Javaid, 2016a; Javaid, 2016b, p. 288; Javaid, 2017; Javaid, 2018; Weiss, 2010). Javaid argues that it is vital to recognize male victims in order to develop a better understanding of the typology of victims, to alert researchers and funders to their marginalized positions and how continued neglect goes towards reinforcing patriarchal power relations and “hegemonic masculinities” (Cohen, 2014; Javaid, 2016b, p. 286). Focusing exclusively on female victims is problematic because rectifying one invisible group (i.e., women) is replaced by making another group invisible (i.e., men). This “may also explain why the status of ‘victim’ does not appear to carry the same credibility for men as for women” (Hine et al., 2020, p. 5). Consequently, there is lack of an equitable provision and access to support services for men; furthermore, while female victims (particularly from minority communities) face barriers to support, men may face additional gender-specific barriers associated with the concept of masculinity and because of the general lack of support services available to them (Hine et al., 2020, p. 7). This article provides much-needed analysis of the typology of abuse and perpetrators that occur in HBV/A and forced marriage cases committed against men.

## Method

### The Case Files

This article utilizes a qualitative research design examining the thematic content of 29 Case Files obtained from The Elm Foundation, a gender-neutral domestic abuse charity in Derbyshire, the United Kingdom, that caters to both female and male victims. The Elm was chosen for several reasons, including: (a) the author formerly served on the management board as a trustee for two years and so was able to gain access to Case Files, (b) The Elm Foundation handles a substantial volume of cases and a variety of calls each year. Between September 2019 and September 2020 (i.e., after the completion of the author’s fieldwork), The Elm addressed 2,310 cases from both female and male victims; 230 of those cases (i.e., 10% of the workload) involved all types of male victims across all services, including refuge and outreach; 9 of those cases involved male victims of HBV/A and forced marriage who then went into The Elm’s refuge (although The Elm had to decline a further 8 of these cases because of lack of bed space—these cases were then passed onto other agencies); and they received 17 referrals of cases from all types of victims by government agencies, including the police, councils and other domestic abuse charities in the country, and (c) The Elm Foundation provides services to victims, as well as to other specialist organizations, including counseling, therapy, refuge space, training, and legal services. This places The Elm in a rare position within the sector itself because they are only one of nine refuges dedicated solely to male victims alone in the United Kingdom (Melley, 2018). This research, therefore, provides a unique opportunity to look into the lives and experiences of male victims; as previous research in this area has primarily focused on female victims, with respect to the concept of diversity, this article argues that the experiences of all victims, regardless of sex or gender, must be explored.

The 29 Case Files relate to intervention and support directly from The Elm Foundation and its male refuge between 2010 and 2019. The 29 Case Files are not the total number of male victims accessing or supported by The Elm Foundation—other types of male victims, including male victims of general domestic abuse, sexual assault, or modern-day slavery with no HBV/A or forced marriage) were excluded from the study. Of the 29 male victims, 8 were British-born Pakistanis; 2 were Pakistani nationals with “no recourse to public funds” (hereafter NRPF); 3 were British-born Bangladeshis; 3 were British-born Syrians; 2 were British-born Somalians; 1 was a British-born Iraqi; and 1 was a British-born Iranian. There were also five White-British male victims of HBV/A; two Romanian nationals, one Lithuanian national, and one male victim from the Gypsy/Traveling community. The youngest male victim was 16 years old, while the oldest victim was aged 54 years old. Overall, the number of victims below 20 years of age totaled 14; the number of victims between 20 and 29 totaled 13; while the remaining two male victims were 43 and 54 years old. None of the male victims were 30–39 years old. The average age of the male victims was 21 years of age.

### Materials, Procedure, and Data Analysis

All male participants provided the requisite consent to use their data for the purposes of this project. The author read, analyzed, and interpreted Case Files through thematic/content analysis in order to acquire a better understanding of experiences, as recorded by refuge staff, who then inputted data on the OASIS Case Management System (Reinharz, 1992, p. 145). This method of analysis concerns identifying common threads, trends, and patterns found across the Case Files and offers an insight into men’s experiences of victimization. This included the systematic reading and observation of themes within the texts, which were assigned “codes” to indicate the presence of qualitatively important content, including “who” abused male victims, “what” type of abuse victims experienced; and the reasons behind “why” they were abused. Thematic/content analysis was not aided by IT software in this study. Instead, the author manually read and coded Case Files by hand, highlighting extracts, developing themes, and creating a thematic map. Notes of themes were kept throughout the fieldwork to ensure reliability, consistency, and a paper trail from the raw data to the final presentation of results.

The thematic/contextual nature and interpretation of files were enhanced with the help of the CEO of The Elm, who had intimate knowledge of each of the male victims, and who had been directly involved in the support of some of the cases, and who understood the challenges associated with their intervention. Furthermore, the broad coverage and long time span of the database meant that refuge staff was able to record many events within many different social settings, providing a rich account of experiences and the gender power relations that existed in each of the male victims’ accounts (Yin, 2003). HBV/A and forced marriages are vastly underreported and it is very difficult to obtain access to male participants who are willing to engage with a qualitative research design. The author decided not to conduct interviews with male survivors on this occasion, or victims who were currently going through intervention—obtaining historic case files with the requisite permission was considered appropriate for this article and The Elm was able to provide data that did not necessitate male victims taking part in interviews. The names of the men have been anonymized in order to protect their identity and privacy.

## Results

### Types of Abuse

Of the 29 Case Files, 16 cases concerned HBV/A and 13 cases concerned forced marriage of male victims (including attempts). Given the interrelationship between the HBV/A and forced marriage, forced marriage will almost inevitably involve elements of “honor,” while not every HBV/A case will necessarily involve a forced marriage (meaning that all 29 Case Files should conceptually be classified as “honor-based”). However, the abuse that the men experienced varied and included physical violence; coercive control; threats of violence because of the discovery of (homo)sexuality; psychological abuse; emotional blackmail; financial control; withholding of food; false imprisonment; being drugged; and being silenced. An archetypal case of HBV/A against men can be found within Case File 16, where a 16-year-old British-Pakistani male was provided with support by an Independent Domestic Violence Advisor (IDVA) service following a referral to this high-risk service, due to violence he experienced from his parents. The victim had been in trouble with the police and the family felt he had brought “shame” upon the family (the victim had a criminal history of stealing cars and had lived a life of crime). His family, in particular his mother and the wider community, requested that he be sent back to Pakistan in order to teach him his “cultural values” and to put him on “straight and narrow.” Community leaders were so concerned that he was a “bad influence” to other young people that they wanted him out of the country. In Case File 21, the 19-year-old British-Pakistani male victim sought refuge with The Elm. The victim’s family felt that he had brought “shame” upon the family because of a relationship he was having with a White-British woman whilst attending university. While the family wanted him to end his relationship, the victim wanted his relationship to continue. Subsequently, his White-British girlfriend was kidnapped by relatives/community members, dragged into a car, and told to leave him alone. She was released shortly after without harm. However, the male victim loved his girlfriend and continued with his relationship, entering the refuge to escape further abuse and threats.

This account is interesting because it also involves “indirect” violence, exposing a third party (i.e., the girlfriend) to intimidation in order to apply pressure on the son to conform.

In relation to forced marriage, in Case File 8, the 17-year-old British-Bangladeshi male experienced an attempted forced marriage as a result of his criminal and perceived promiscuous behavior. He was due to board a flight to Bangladesh from Heathrow airport. On the morning of the flight, his parents laced his breakfast with drugs and he was comatose throughout the journey to the airport. On arrival, the effect of the drugs wore off and the victim said he wanted to go the toilet, upon where he made his escape out of the airport. He later contacted an outside agency with the support of friends, who then referred him to The Elm. Similarly, Case File 2 revealed a 17-year-old British-Pakistani male who experienced an attempted forced marriage after his parents suspected he was gay—he experienced physical and psychological abuse in an attempt to marry him off. Six attempts were made by the police before removing him into The Elm’s male refuge. The victim suffered severe depression and attempted suicide several times whilst in refuge. In total, nine of the male victims (Case Files 2, 4, 10, 11, 13, 14, 20, 25, and 29; 31% of the Case Files) experienced HBV/A and/or forced marriage specifically because of the discovery of their (homo)sexual orientation.

However, not all of the forced marriage cases involved criminal or promiscuous behavior, or the discovery of homosexuality on the part of the male victim. Some male victims exhibited “conformist” behavior but were still subjected to forced marriage. In 11 of the Case Files (Case Files 3, 6, 9, 15, 17, 18, 22, 23, 24, 27, and 28; 37.9% of the Case Files), heterosexual men were also attempted/forced into marriage to fulfill planned promises to marry relatives in the United Kingdom or abroad, with perpetrators wishing their heterosexual sons to marry relatives of the perpetrators’ choosing. In Case File 6, the forced marriage was actually undertaken, with the male taken on a “family holiday” with no discussion or warning that a marriage was going to take place until he had arrived in Pakistan. In Case File 27, IDVAs supported a 22-year-old British-Bangladeshi male victim attempting to resist a forced marriage. The victim rang the FMU helpline explaining that he was frightened that he was going to be taken abroad. IDVAs met him at the doctors (it was a planned meeting with the doctor’s knowledge) and they planned to help him leave home. Elements of trickery were also employed by his family—they had “promised” to return to the United Kingdom if he entered the marriage. Through The Elm’s intervention, the male victim was rehoused and supported by the FMU.

Three of the male victims (Case Files 5, 12, and 19; 10.3% of the Case Files) with physical and learning disabilities were also targets of HBV/A and/or attempted forced marriage because families believed that to be the best way to support them in the long term, confirming research by Clawson, Fyson, and others that these vulnerable men are equally at risk of abuse as women (Clawson, 2016; Clawson & Fyson, 2017; Rauf et al., 2013).

### Men Harmed by Patriarchal Figureheads

There were a variety of perpetrators involved in both the HBV/A and forced marriage of male victims, but most of the main perpetrators were fathers and senior male figureheads in the family. This was followed by mothers, families in general, extended families, in-laws; wives; and intimate partners (including some perpetrators in same-sex relationships). The data revealed the nature of power and control that existed between the male victims and their male perpetrators. Male victimization occurred in these cases because patriarchal figureheads (mainly fathers in nearly all of the accounts: Case Files 1, 2, 3, 4, 5, 6, 8, 9, 10, 11, 12, 13, 17, 18, 19, 20, 21, 22, 23, 24, 25, 26, 28, and 29; 82.7% of the Case Files) sought to exercise power and control over their younger male children. For example, in Case File 17, the IDVA service supported a 23-year-old British-Pakistani male following a dispute with his family over an “arranged” marriage. The male victim had wanted to go to university, but his father rejected this. The male victim resisted all attempts at the “arranged” marriage and his situation quickly escalated to a “forced” marriage. In the Case File, the male victim stated that he could hear his father plotting about him and making plans for him to get married. The father also made numerous claims and promises, but the male victim did not believe him and thought his father was trying to trick him in order to trap him. With the support of The Elm and IDVA service, the male victim was supported to live outside the area where his family resided, with friends that he had made at university.

### Female Perpetrators

Fourteen Case Files (Case Files 2, 3, 4, 5, 6, 8, 9, 11, 13, 18, 20, 21, 25, and 28; 48.2%) reveals what appears to be the perpetration of abuse by mothers as secondary agents, supporting the abuse committed by fathers and patriarchal figureheads. However, Case Files 1, 7, 14, 15, 16, and 27 (20.68% of the Case Files) involved abuse by mothers and wives who were very clearly described as “controllers.” In Case Files 15, 16, and 27 (10.34% of the Case Files), mothers were the direct instigators of the attempted forced marriage of their sons (and not fathers) and were labeled as the main and direct perpetrators of the abuse of their sons.

Case Files 1, 7, and 14 (10.34% of the Case Files) revealed the “complex,” “multi-faceted,” and multi-dimensional features some domestic abuse cases present, where it is perfectly possible for a case that starts out as domestic abuse to then transform into HBV/A. In Case Files 1 and 7, the Pakistani-born male victims were seriously abused by their wives (along with their in-laws and other family members). They were abused on the basis of their immigration status, perceived as lower class, and were treated like modern-day slaves, demonstrating the multiple positions that men occupy. There was a clear power order within the relationships and in Case File 1, the wife and her brother were categorized as direct perpetrators and the main instigators of abuse. Although it is not always clear that abuse in these types of cases is always HBV/A, when the two male victims in Case Files 1 and 7 contacted their families back home in Pakistan asking for intervention, they were emotionally blackmailed to remain within their marriages for the sake of familial “honor.” This, then, transformed their experiences from one of domestic abuse into HBV/A—the male victims were silenced to protect the “honor” of their natal family as well as their in-laws. When the in-laws were made aware that the male victims had contacted their families back home for support, they were then subjected to further acts of abuse, control, and imprisonment to silence them from speaking out. This is similar to the Pakistani-born women in Idriss’s PhD thesis, who sought intervention from their families back home against their husband’s and mothers-in-laws’ domestic abuse, only to be told that they could and would not help (Idriss, 2018b).

In Case File 1, the male did not even recognize that he was a “victim”—he was over 6 feet tall, while his wife was considerably shorter; he had never even heard of a male refuge. In Case File 7, the wife was so incensed by her husband’s eventual departure that she called the police alleging that he was a terrorist planning a major attack in London in order to facilitate his deportation back to Pakistan. She, and her family, were so “dishonored” by his departure that they tried to shift the blame on the marriage ending on (false) allegations of terrorism, rather than their own abusive and controlling behavior (the allegations of terrorism were investigated by the police before being dropped, with the aid of The Elm).

### HBV/A in White (European) Communities

An interesting finding not discussed within the existing literature is HBV/A within White majority/British communities and same-sex HBV/A. Case Files 4, 10, 11, 12, 13, 14, 19, 25, and 29 (31% of the Case Files) specifically demonstrate that HBV/A can occur in White Christian, European communities (i.e., the male victims were from British, Lithuanian, Romanian or Gypsy Traveler backgrounds). It confirms that HBV/A is not specific to one culture or group, but affects victims from a wide range of ethnic backgrounds (Idriss & Abbas, 2010). In Case File 4, the White-British victim and his White-British boyfriend (the main perpetrator) were both in a homosexual relationship. When the victim wanted to end the relationship on account of the perpetrator’s coercive, controlling, and domestic abuse, the perpetrator “outed” the male victim’s sexual orientation on social media, knowing very well that this was a source of “shame” for the victim and “dishonor” for his family. This consisted of a mixture of both direct (the “outing” itself) and indirect abuse (the risk of abuse from the victim’s family). In the Case File, the victim described that his father once told him that “he would put a bullet in his head if he was gay and that he would beat the gay out of him” (n.b. these same words were also uttered by the father of the victim in Case File 20, a 17-year-old Somalian Muslim male who came out as gay). The boyfriend in Case File 4 was aware of the victim’s father’s homophobic views and deliberately outed him on social media in order to cause him problems and as an act of revenge for ending the relationship. The threats of violence from the male victim’s family members (whether or not carried out) made them potential perpetrators of HBV/A, but the boyfriend who outed the male victim on social media can also be considered to be a perpetrator (even if only vicariously), given that he knew his revelation on social media would be a source of “shame” and was aware of the consequences involved for the victim.

In Case File 13, the victim was a White-British 17-year-old from the gay community. He was forced out of his home in his underwear and subjected to violence by both his father and brother. The brother had outed the victim to his parents as he had seen something on social media that gave an indication that the victim was gay. The family felt this was a source of “shame” as the father was an active member of English Defence League (EDL), a known far-right group that opposes Islam, Muslims, and homosexuals. The victim was then taken into social care in a children’s home and provided specialist support in a care home with The Elm’s intervention. Similar facts occurred in Case File 29, where the father of a 21-year-old White-British male discovered his homosexuality. His father was also very “right-wing” and became very violent and ashamed by the fact that his son was gay.

In Case File 14, the White-British 54-year-old male came out as gay following the end of his heterosexual marriage. His wife’s family then became very violent towards him because they were concerned that the family would be ridiculed if knowledge of his sexuality became public (i.e., that the children would be teased at school). Although not proven, it was alleged that the wife’s family committed arson as his property was set on fire. The victim experienced HBV/A and financial abuse from his wife, when money went missing and joint bank accounts were closed without his knowledge. Similarly, in Case File 12, the male victim entered The Elm’s male refuge because of abuse by his parents. His White-British mother and father were devout Catholics. The male victim was gay and the father had discovered that the victim had liked to dress in women’s clothing. The parents were so ashamed by this discovery that they took him to Church to be lectured by the Priest so that he could “cure his gayness” and of his preference to wear women’s clothing. Later, his parents changed the locks of the home and disowned him; they also stopped his income stream as the male victim was financially dependent on the father. The victim soon after entered The Elm’s male refuge.

## Discussion

### Men Harmed by Patriarchal Figureheads

This article aims to identify how and why men are harmed in HBV/A and forced marriage cases, exploring both the similarities and differences in the ways that men, in comparison to women, are harmed by patriarchy. Male victimization occurred in these cases because fathers had sought to exercise power and control over their sons. This is interesting because in other research, other male figureheads such as uncles have been responsible for abuse (Bates, 2017). Patriarchal theories of violence therefore apply to male victims just as much as they do to female victims. There are common strands and very strong analogies between the various reasons why HBV/A and forced marriage are committed against women and the men in this study. This namely relates to sexual deviancy, which has the same basic premise for both sets of victims and the same types of responses from their families but can generally manifest itself in different ways. For women, it can relate to boyfriends, pre-marital sex, extra-marital affairs, and sexual orientation. For the men in this study, it would appear to mainly relate to their sexual orientation (Bates, 2017, pp. 111–113). There are also different particularities to this assertion. Men are able to get away with being sexually deviant far more than women can—the threshold for men, at least in the context of sexual deviancy, appears to be much higher before men become victims because men are not criticized as much for having sexual relationships outside marriage. As long as they keep their indiscretions hidden, immediate family members may be more willing to tolerate their sexual (mis)behavior (“the boys will be boys” maxim: Mooney, 2008). There is, therefore, some subtle control in these types of cases. This can be contrasted to women, who are controlled more vigorously and harshly. Even allegations or suspicion of sexual misconduct can lead to women experiencing HBV/A, forced marriage, or risk of being killed (Gill et al., 2014).

But while the victim groups in HBV/A and forced marriage cases consist mostly of women, they also consist of the “wrong sort” of man. In this study, fathers were the main abusers who carried out abuse upon their sons who did not conform to gender-role expectations on masculinity, particularly in those cases where the non-conformist sexuality of male victims was discovered. This study provides new empirical data that specific groups of men may be harmed by patriarchs on account of their sexual deviancy or promiscuity on the one hand, or “otherness” (such as disobedience to an arranged marriage and disability) on the other (Rogers, 2017; Samad, 2010). As nine of the male victims experienced abuse because of the discovery of their sexual orientation, this suggests that the threshold of patriarchal tolerance had been reached and abuse was then inflicted. Families wanted to keep their sons’ sexual orientation hidden from the rest of the community. These analogies demonstrate how patriarchal theories similarly apply to both female and male victims in relation to sexual deviancy. In relation to men, institutions such as the family and religion each justify and reinforce young men’s subordination to senior men, resulting in men’s internalization of inferiority if their sexual orientation becomes known because patriarchy constructs homosexual men as “not being masculine enough” (Bates, 2005).

Connell and Mackay explain that all men benefit from patriarchy (unlike women) and so men are still able to take advantage of the “patriarchal dividend,” which includes benefits of authority, respect, access to institutional power, and control over one’s own life (Bates, 2005; Mackay, 2015). Men in general are able to enjoy more freedoms and can control their lives, more so than women—while men are expected to follow strict norms of behavior, they simultaneously have more freedom to violate those norms. This has been written about in relation to the differential impact forced marriages have on men (in comparison to women). It has been evidenced elsewhere that in some forced marriage cases, male victims may then go on to have a “second” wife, an English girlfriend or boyfriend with the tacit approval of family, as long as they conform publicly to the masculine, husband role, and taking advantage of their privileged positions, which women are not afforded with (Chantler, 2020). However, in this study, while male victims may have been born with the potential of benefitting from the “patriarchal dividend,” some lost that benefit through non-conformist gender-role behaviors, failing to conform to understandings of masculinity, and where the man’s homosexuality was discovered. Recent research has highlighted that homosexuality is a big trigger in HBV/A and forced marriage cases for men (Bates, 2020a; Jaspal, 2020; Khan & Lowe, 2020). Although all men begin by benefitting from the “patriarchal dividend,” not all are able to maintain it. The failure to conform to essential norms of masculinity meant that male victims were no longer viewed as “men” and could legitimately be subjected to abuse. These men did not have equal access to power and privilege and did not have an equal investment in the current gender order. There was no obvious advantage of the “patriarchal dividend” for these victims, who were clearly unable to take control over their own lives.

The ways in which male victims were harmed was not particularly different to women, nor were the means used to inflict harm any different to VAWG in similar circumstances (Björktomta, 2019). Upon the discovery of homosexuality, male victims experienced physical violence, coercive control, imprisonment in the home, emotional blackmail, psychological abuse, and attempted forced marriages, bearing some similarity with women who are forced into marriage upon the discovery of (alleged) illicit sexual relationships (Bates, 2018; Samad, 2010). Perpetrators in these cases wanted to control male victims to prevent undesirable (homosexual) relationships, whether or not the victims were in an actual relationship. When they refused to accept patriarchal ideology and commands, senior men then used violence to accomplish what patriarchal conditioning had failed to achieve. Very much like feminist theories used to explain why women become victims of violence, men may react to coercion as a way of bargaining with patriarchal forces and as a means of ensuring their own physical, emotional and psychological well-being (Aplin, 2017; Hunnicutt, 2009). Similar to Kandiyoti’s examination of women’s “bargaining with patriarchy” (Kandiyoti, 1988), the streetwise man understands that if he wants to survive in a patriarchal setting, he had better act “like a true man,” otherwise he may be subjected to harm. This understanding of men’s experiences demonstrates the application of patriarchal theory (or, at least, a variant of it) to male victims. Whether we are discussing women’s or men’s sexual deviancy, these examples almost always results in the same outcome—HBV/A, forced marriages, or so-called “honor” killings, although, with regards to the latter, women are at far greater risk of being killed than men (Gill et al., 2014). Patriarchs were clearly responsible for abuse, which was pursued in order to enforce male dominance, to “correct” and to punish perceived (mis)behavior that contravened patriarchal values and to prevent any further “dishonor.” This is directly analogous to the VAWG position and female victims who are considered to have brought “shame” on their families because of their sexual (mis)behavior. Similarly, fathers may have feared that they themselves would be considered “less manly” by their own cultural norms for not ensuring that their sons conformed to culturally accepted rules, and so enacted abuse (Reddy, 2008). This discussion also demonstrates that both male and female victims are fighting the same battle—patriarchy, hegemonic masculinity, and male domination over those who are weaker. In the HBV/A and forced marriage field (but not necessarily in wider violence), men’s experiences of victimization have more in common with women than previously identified, although there are important differences. While this article does not fully address statistics on prevalence, it is still worth noting that evidence shows that more women than men are victims overall—this is one difference between the two victim groups.

Forced marriage was also used as a tool to curb the perceived wayward behavior of some of the men concerned. In Case Files 8 and 16, HBV/A and forced marriage were used to control the behavior of the male victims and to put them “on the straight and narrow” because of their criminal behavior and promiscuity. In these two case examples, men were controlled and abused for the purposes of protecting or enhancing the family’s reputation. It would appear that having many sexual partners challenged the current order and a masculine response was used to make the male victims look weak. The victims were “too male” and so they became victims of HBV/A or an attempted forced marriage as a way to control them. The focus, therefore, is not exclusively on “deviant” masculinities—despite the “boys will be boys” maxim, men who are too sexually active may also experience victimization if such men are deemed too “wayward” or “uncontrollable.”

However, heterosexual men were also forced into marriages against their will for the sake of familial “honor” in order to fulfill planned promises to marry relatives in the United Kingdom or abroad. To then retract a promise of a marriage was perceived by perpetrators to bring “shame” upon the family. The aim of such marriages is to strengthen family ties, to assist in claims to residency in the United Kingdom (Idriss, 2015), as well as to ensure that cultural and religious values are passed onto the next generation (Cressey, 2006). Like female victims, these male victims were objectified through depriving them of their free choice and independence (Keyhani, 2013). “Honor” within the context of forced marriage is viewed as a form of “social currency” and “property”—the giving away of an individual for marriage means exchanging the family’s izzat and respect (Bond, 2012; Bond, 2014), which tends to be seen as a “family” rather than an “individual” affair (Dale & Ahmed, 2011). Heterosexual victims were then abused if they “disobeyed” orders to marry—their actions challenged the patriarchal order and awakened anger in their perpetrators.

### Links to the “Body” as With Women?

This article has so far explained and developed a variety of theories in the area of HBV/A and forced marriage studies. First, it demonstrates that patriarchy is an important causal factor in the abuse of men and that patriarchal theory development must now properly recognize male violence against women and men. Men’s experiences of victimization are very similar to women’s in other ways too. “Honor” may also be linked to the male “body” when men exhibit non-conformist sexuality—in such cases, patriarchs may then attempt to “defend” the family’s “honor” by inflicting various forms of abuse. It will be recalled that nine of the Case Files concerned male victims who had experienced abuse because of their sexual orientation. The male in Case File 2 was forced into marriage on account of his homosexuality and the issue of homosexuality in South Asian communities represents an expectation that families monitor their members to ensure that “honor” norms are obeyed (Lowe et al., 2019). Given the patriarchal nature of collectivist cultures like South Asian and Muslim communities, rigid gender norms guide social behaviors’ in terms of what is considered appropriate behavior for both men and women. Men are expected to demonstrate strength and sexual power that endorse traditional masculine values, yet homosexuality is perceived to violate those traditional gender norms, explaining why the nine men experienced anti-gay abuse. When discussing male victims and their sexual orientation, there appears to be a similar link to women’s victimization and to the “body” as is often discussed in radical feminist literature.

The “shame” of revealing one’s sexuality explains why homosexual men are reluctant to disclose their sexuality, and in particular, to their fathers. Social ideals on heterosexuality and masculinity, often underpinned by religion, dictate that men are the “penetrator,” not the “penetrated,” and are the “pursuers of sex,” rather than “pursued” (Javaid, 2016b; Weiss, 2010). Within this conception, women are the pursued and are penetrated by a penis, yet a man who discloses his homosexuality (and of his desire of being the penetrated, as well as the penetrator) undermines norms of heterosexuality and masculinity (Javaid, 2016b; Weiss, 2010). Norms on heterosexuality create social constructions and heterosexual relationships are usually constructed with regards to penetration. Heterosexuality is understood with reference to whether bodies penetrate or are penetrated. Anal penetration is considered “unnatural” or “abnormal”—for a man to be penetrated by another man violates cultural perspectives on hegemonic masculinity (Javaid, 2016b; Weiss, 2010). Butler elucidates this point by suggesting that the “feminine” is always the penetrated, while the masculine is always the impenetrable (Butler, 1993, p. 50).

Although the general understanding may be that homosexual men want to be penetrated, a homosexual couple will still require a penetrator. The cultural stereotype is that of a homosexual man being penetrated and this is what violates these cultural perspectives, regardless of whether the victim of abuse is the penetrator, the penetrated, or a mixture of both. Hegemonic masculinity dictates that men are not supposed to desire a penis and the discovery of a man’s homosexuality can lead to “shame” because social constructions expect men to be manly and to satisfy women’s sexual desires, a conception reinforced by contemporary pornography (Javaid, 2016b; Weiss, 2010). In the Case Files, it was acceptable for fathers to act with verbal and physical aggression against their sons on account of their sexuality, confirming that the patriarchal order prohibits homosexual relationships as such men do not meet established expectations on hegemonic masculinity (Bates, 2005, pp. 78–81, 85, 143–163). Furthermore, these nine Case Files (and by extension, Case Files 8 and 16) demonstrates that patriarchal figureheads, within a religious and cultural setting, always seem to cast one eye over junior men and on their sexual behavior—men therefore cannot act with complete freedom—and if there is any inclination that a son might be gay or is acting “out of control,” the male “body” is then viewed as a potential source of “shame.” This was expressly referred to in Case Files 4 and 20 (where threats to kill by the two fathers for being gay demonstrates the analogies to female victims, as there was an express focus on the male “body”—“beat the gay out of him”). Similar to women whose sexual (mis)behavior may devalue the social capital of the family (Bond, 2012; Bond, 2014), gay and “out of control” men are controlled in order to safeguard the family’s “honor.” This includes forcing such men into (heterosexual) marriages against their will in order to cast away any negative aspersions that may be made. Here lies the patriarchal focus and attention on the male “body,” as with the female “body,” perhaps in more ways than the existing literature acknowledges. As a specific group consisting of male victims, it is therefore unsurprising that gay men are reported to be at greater risk of HBV/A and forced marriage from their immediate and extended families (Björktomta, 2019; Lowe et al., 2019; Rogers, 2017). However, sleeping around, “dishonoring” too many women and criminal behavior (e.g., Case Files 8 and 16) could still potentially create a bad reputation for the family and therefore make some men less marketable for marriage proposals. It would appear that doing something with the “body,” whether it concerns promiscuity, homosexuality, lesbianism, transgenderism or sexual deviancy in general, and which contravenes culturally accepted norms as set by the patriarchy, can lead to HBV/A and forced marriages, whether the victim is female or male.

### Typology of Abuse

This article also supports L. Bates’s typologies of HBV/A and forced marriage cases. As part of her study, she devised three typologies of HBV/A, primarily defined by perpetrators and their relationship to the victim. Type 1 involves abuse from a current or former intimate partner, where “honor” is used as an explicit tool of control in a one-to-one relationship (Bates, 2017, p. 239; refer also to Bates, 2020b). Type 2 typically involves abuse from the victim’s natal family members, usually parents, sometimes acting together with others including (but not limited to) brothers, aunts, uncles, and cousins (Bates, 2017, p. 240). In such cases, “honor” operates as natal family pressure to marry or otherwise comply with behavior or lifestyle that the family wishes, based on traditional gendered family roles (Bates, 2017, p. 240). Type 3 abuse typically involves abuse from a current or former intimate partner acting with other family members (usually the victim’s in-laws) and relates to gendered generational roles. It is clear that these three typologies apply to women as victims, whether as daughters, sisters, wives, or daughters-in-law. However, this article shows how male victims also fit within the typologies devised by Bates as part of an overall understanding of patriarchal violence—that young, weaker men may experience HBV/A and how it seeks to deter young men from expressing themselves. It is worth noting that L. Bates’s study applies to both female and male victims (as it does perpetrators) and she discusses the application of the typologies to male victims, whilst raising some distinctions (Bates, 2017, pp. 111–113). L. Bates also states that Type 1 does not seem to exist for male victims; Type 2 is similar for both male and female victims; and Type 3 exists for both sets of victims, where they are victims of abuse from in-laws when a relationship ends, but her distinction is that a man leaving brings shame to his wife (for which his in-laws then abuse him), whereas for female victims, it tends to arise from the male’s family wholly rejecting her as “inferior.”

This article extends L. Bates’s work on applying the typology to male victims by providing new evidence on how the dynamics of HBV/A work for men and linking this within wider masculinity theory. Applying the typology to the current dataset of 29 Case Files, one case exhibited Type 1 abuse (Case File 4; 3.4% of the Case Files), where the male victim was abused by a former intimate partner and where “honor” was used as an explicit tool of control in a one-to-one relationship. However, this occurred in only one case and it is suggested that this form of abuse from an intimate partner, while it exists, is rare for men to experience, while Type 1 abuse appears to be very common for women (Bates, 2017, p. 239). Again, this is another distinction between men and women. Admittedly, traditional definitions of HBV/A involve family, community members, and other social groups who may be perpetrators (Her Majesty’s Inspectorate of Constabulary, 2015), but in relation to Case File 4, this new and emerging type of case can also be considered to be HBV/A, but includes a perpetrator like a boyfriend who would appear to fall within L. Bates’s Type 1 typology. Given that Type 1 involves abuse from a current or former intimate partner and where “honor” is used as an explicit tool of control in a one-to-one relationship, Case File 4 appears to fall within Type 1 in terms of developing typologies of perpetrators and male victims. State agencies and policymakers may need to revisit the definition of HBV/A to make it clearer and take into account other types of perpetrators, like the one in Case File 4, who arguably do not fall under obvious “family,” “community,” or “other social groups” (Idriss, 2017). While Case File 4 may be considered Type 1, the dynamics of the White-British same-sex relationship and homophobic shaming adds a particular angle to the area of HBV/A which has not been discussed within the existing literature (Bates, 2017).

However, 25 case files (86.2% of the Case Files) exhibited Type 2 abuse (Case Files 2, 3, 5, 6, 8, 9, 10, 11, 12, 13, 15, 16, 17, 18, 19, 20, 21, 22, 23, 24, 25, 26, 27, 28, and 29). Thus, the vast majority of the sample typically concerned Type 2 abuse from the male victim’s natal family members (primarily and overwhelmingly fathers, but often in collaboration with mothers as secondary perpetrators), where “honor” operated as natal family pressure to marry or otherwise comply with behavior or lifestyle that the family commanded. It is suggested that Type 2 abuse is more commonly experienced by men; this is also supported by L. Bates’s findings (Bates, 2017). Only three male cases exhibited Type 3 abuse (Case Files 1, 7, and 14; 10.3% of the Case Files), typically involving abuse from a current/former intimate partner acting with other family members (i.e., the in-laws). Again, although not very common in the current sample, Type 3 abuse appears to be more common than Type 1 abuse for men. This discussion also demonstrates the clear overlaps between male and female victims—both sets of victims can fall into either Types 1, 2, and 3 abuse.

### Female Perpetrators—Controllers, Collaborators, or Coerced?^1^1.These are terms borrowed from Bates’s (2018) paper on “who is involved” in “HBVA” cases. The “Conclusion” section also draws on the suggested “three roles” of female perpetrators within that paper, particularly p. 294 onwards.

Fourteen out of the 29 Case Files revealed what appears to be the perpetration of abuse by mothers as secondary agents. Women who are “collaborators” on behalf of patriarchs can be a difficult subject to broach (Gangoli, 2007, p. 50), given that the traditional focus in the literature is on VAWG. Women’s violence against men within interpersonal relationships specifically remains an under-researched and under-developed area and E. Bates et al. highlight the urgent “need for an inclusive research approach that recognizes men’s victimization” in domestic abuse cases in general (Bates et al., 2019). It has already been stated that there is scant research on men’s victimization, but another distinction that can be drawn here is that there is even less discussion on women’s violence against men specifically in HBV/A and forced marriage studies. This is probably the reason why female perpetrators of male victims have received so little attention within the existing literature (Aplin, 2017).

Pope states that the role of women in keeping harmful practices alive is perplexing—though patriarchal rules are designed to serve the needs of men, women are often patriarchy’s most enthusiastic agents because of survival and the need to avoid being harmed (Bates, 2018; Pope, 2012). Women may become involved in the policing of others because they themselves are controlled and oppressed—they then perpetrate abuse on behalf of the patriarchy. In the Case Files, there was lack of evidence on the specific role of female family members, in particular, of their own personal motivations in the involvement behind abuse. Nevertheless, the evidence suggested that some of the mothers acted—in L. Bates’s terminology—as “collaborators” (Type 2) against their sons, so one could hypothesize that these women were subjected to threats and coercion, nor afforded with the agency to go against their more powerful husbands. Women will make choices within particular contexts and this includes bowing down to patriarchal pressures or doing “patriarchy’s dirty work” (Card, 2000, p. 513). Some may have rationalized that challenging the established patriarchal order would be futile as well as dangerous because they themselves might have become targets—by “bargaining with patriarchy” they are at least granted a small share of power (Pope, 2012, p. 103; Kandiyoti, 1988).

However, a counter-argument is that mothers were not expressly described as “secondary victims” within the Case Files. It is possible that some mothers internalized patriarchal ideology, collaborating in abuse without any coercion, by buying into dominant socio-cultural norms and traditions where, for example, homosexuality is considered wrong. This could make them “controllers” in L. Bates’s terminology. Alternatively, not recording mothers as secondary victims may have had more to do with staff at The Elm seeking to support the transition of male victims through refuge, rather than trying to analyze who else might be “victims” within the home. Further investigation would be needed to determine the reasons why mothers took part in the HBV/A or forced marriage of their sons.

Six out of the 29 Case Files, on the other hand, involved abuse by mothers and wives who were very clearly described as “controllers,” in L. Bates’s terminology, and who were direct perpetrators of abuse against their sons and husbands. In Case Files 15, 16, and 27 (10.34% of the Case Files), mothers were the direct instigators of the attempted forced marriage of their sons. This challenges traditional assumptions that present women as subordinate and passive (Aplin, 2017, p. 9; Grady, 2002, pp. 71–96). In Case Files 1 and 7 (concerning the abusive wives), the wives were well aware that what they were doing was wrong. Being the defenders or guardians of familial reputation, it would seem, is not exclusive to men and is no different to those cases that involve female-on-female HBV/A as in Idriss’s PhD thesis (Idriss, 2018b). The abuse by the two wives in this study had to be hidden at all costs—it was the shameful behavior of the wives and in-laws that had to be hidden; the male victims did nothing wrong and were totally blameless. Viewing all women as homogenously powerless is not only disrespectful to male victims, but it does not provide for a sophisticated theorization of women who are direct “controllers” and who become the benefactors of oppression for their own individual reasons.

## Conclusion

Although women are overwhelmingly the main victims in HBV/A and forced marriage cases, male victims in this field can no longer be ignored. Like women, men are harmed by patriarchy and by the same kinds of perpetrators; there are also striking analogies in the ways in which both women and men are victimized in natal violence. The data in this article demonstrates the importance of properly recognizing male victims and the various perpetrators these types of cases present, including fathers and the involvement of mothers as primary or secondary perpetrators. Until academic research on, and consequently service provision for, male victims is taken more seriously, men will not come forward and disclose abuse, exacerbating the belief that “real” men cannot be victims. A gender-inclusive conceptualization of “victimhood” needs to take into account the various factors that contribute to victimization, for both women and men, in order to understand both the similar and different reasons why they are abused. Recognizing the differences and the impact intervention has for men will also help to encourage tailored responses that are better suited for male victims. State responses are more formalized for women when it concerns VAWG as a result of improved awareness and campaigns; practical responses, however, are less formalized and developed for men. There is wider significance to this research for male victims, for all victims in general, and for state intervention to be more coordinated and proactive when male victims enter the system. There should be separate government policy on Ending Intimate Violence Against Men and Boys and separate funding for Ending Violence Against Men and Boys that mirrors policies for female victims of violence, in order to address the imbalance that currently exists in the provision of support services.

## Limitations

There are some limitations to this study. One relates to the accuracy of the Case Files, for they may not be accurate and may include bias in themselves, colored by the narrative accounts of the victim’s story as written by caseworkers (Yin, 2003, p. 87). This is because Case Files represent inserted data, interpreted and entered into the system by the relevant caseworkers at the time. Case Files may also be subject to individual bias of the (multiple) people who had authored them. They must be carefully used and should be treated with caution. An obvious limitation also relates to the sample size, which may not be representative or reflective of the types of cases other organizations have encountered. The author does not underestimate the significance of these limitations, but this study provides an important snapshot of 29 Case Files that were sampled at a particular moment in history and time. The author is mindful that there may be other reasons, factors, and explanations why men may experience HBV/A and forced marriages. However, whilst acknowledging these concerns, this study provides a strong original contribution and an important glimpse into the lives of 29 male victims of HBV/A and forced marriage, and the reasons behind their abuse.

## Author Biography

**Mohammad Mazher Idriss**, LLB (Hons), LLM, Cert Ed, PhD, is a senior lecturer in Law at Manchester Metropolitan University. He completed his PhD thesis in 2018 on “Honour-based Violence in Ethnic Communities in England and Wales,” which was externally examined by Professor Lynn Welchman (of SOAS, University of London). He is the author of a number of co-edited books and articles. His most recent publications (as chief editor) include: Men, Masculinity and Honour-based Abuse (Routledge-Cavendish, 2020); “The mosques are the biggest problem we’ve got right now:” Key agent and survivor accounts of engaging mosques with domestic and honor-based violence in the United Kingdom” (2020, Journal of Interpersonal Violence, 35(13–14), 2464–2491); “Key agent and survivor recommendations for intervention in honor-based violence in the UK” (2018, International Journal of Comparative and Applied Criminal Justice, 42(4), 321–339); “Not domestic violence or cultural tradition: Is honour-based violence distinct from domestic violence?” (2017, Journal of Social Welfare and Family Law, 39(1), 3–21); “Criminalisation of the burqa in the UK” (2016, The Journal of Criminal Law, 80(2), 124–137); “Forced marriages: The need for criminalisation?” (2015, Criminal Law Review, 9, 663); and “Sentencing guidelines for HBV and honour killings” (2015, Journal of Criminal Law, 79(3), 198–210). He is also co-editor (with T. Abbas) of Honour, Violence, Women and Islam (Routledge-Cavendish, 2010).
